# Dynamic MRI for bowel motility imaging—how fast and how long?

**DOI:** 10.1259/bjr.20170845

**Published:** 2018-03-03

**Authors:** Catharina S de Jonge, Ruaridh M Gollifer, Aart J Nederveen, David Atkinson, Stuart A Taylor, Jaap Stoker, Alex Menys

**Affiliations:** 1 Department of Radiology and Nuclear Medicine, Academic Medical Center, Amsterdam, Netherlands; 2 Centre for Medical Imaging, University College London, London, UK

## Abstract

**Objective:**

Dynamic imaging of small intestinal motility is an increasingly common research method to examine bowel physiology in health and disease. However, limited data exist to guide imaging protocols with respect to quantitative analysis. The purpose of this study is to define the required temporal resolution and scan duration in dynamic MRI for small bowel motility assessment.

**Methods:**

Six healthy volunteers underwent motility imaging with MR enterography using breath-hold protocol. A coronal two-dimensional balanced fast field echo sequence was used to acquire dynamic data at a high temporal resolution of 10 frames per second (fps). Motility was quantified by generating a registration-derived motility index for local and global regions of bowel. To evaluate temporal resolution and scan duration, the data were undersampled and the scan length was varied to determine the impact on motility index.

**Results:**

The mean motility index stabilizes at a temporal resolution of 1 fps (median absolute percentage change 1.4% for global and 1.9% for local regions of interest). The mean motility index appears to stabilize for scan durations of 15 s or more in breath-hold (median absolute % change 2.8% for global and 1.7% for local regions of interest).

**Conclusion:**

A temporal resolution of at least 1 fps and a scan duration of at least 15 s is necessary in breath-hold scans for consistent motility observations. The majority of small bowel motility studies to date are in line with these requirements.

**Advances in knowledge:**

This study suggests the minimum temporal resolution and scan duration required in breath-hold scans to obtain robust measurements of small bowel motility from MRI.

## Introduction

Intestinal motility is an essential physiological process that moves food through the gut. Deranged motility, however, is common in disease and aberrant motility is implicated in numerous functional gastrointestinal disorders.^1^ For example, hypomotile segmental contractility in Crohn’s disease is a powerful biomarker of inflammation,^2–5^ pan-enteric changes are seen in neuromuscular conditions such as chronic intestinal pseudoobstruction^6, 7^ and changes in the co-ordination of contractile activity likely underpin common conditions like constipation.^8^ Use of MRI to explore intestinal dysmotility has rapidly expanded, in part driven by increased clinical uptake of MRI enterography in evaluating small bowel disorders,^9^ coupled with advances in post-processing technologies^10^ enabling rapid and reliable quantification.^11, 12^ Indeed, MRI-quantified bowel motility is providing new insights into the importance of aberrant gut motility in disease.^13–17^

To date, most of the research into bowel motility quantification using MRI has made assumptions regarding acquisition protocols. These assumptions concern (1) temporal resolution of image capture and (2) duration of acquisition, neither of which has been rigorously tested. Many researchers acquire motion capture sequences at 1 image s^-1^, typically over a breath-hold of ± 20 s, yet the rationale for this has not been firmly established. With respect to temporal resolution, the literature suggests that the small bowel undergoes between 9 and 12 contractions per minute, and this so called slow wave activity is described to be continuous and regular in the fasted state.^18^ However, Menys et al^12^ using 20 s breath-hold dynamic MRI data showed regions of bowel that were almost static even in healthy subjects. It is assumed that acquiring images at 1 image s^-1^ is sufficient to resolve small bowel contractions, although the implications of inadvertently undersampling contractions are significant, and again this assumption has yet to be formally established.

A 20 s scan duration is usually chosen for pragmatic reasons; coinciding with the amount of time a patient is able to hold their breath during scanning. However, due to a lack of periodicity in contractions, this could lead to inconsistent results and inaccurate conclusions drawn from bowel motility measures. Alternatively, and importantly for clinical practice, 20 s might be too much time, and a saving in scan duration could potentially make cine imaging a more efficient addition to clinical workflows.

In this study, we implement an accelerated 10 images s^–1^ two-dimensional MRI sequence acquired during a breath-hold. This protocol was chosen to “over-sample” bowel motility and quantify the small bowel motility using a registration based technique to provide guidance on the required temporal resolution and scan duration for consistent small bowel motility assessment.

## Methods and materials

### Volunteers

Six healthy subjects (median age, 22 years, range, 21–25 years, 3 females) were recruited prospectively by advertisement and interview. Inclusion criteria included healthy, human volunteers who were willing to undergo minimal bowel preparation and MRI. Exclusion criteria were contraindications to undergo MRI, age younger than 18 years or older than 45 years, history of abdominal surgery, gastrointestinal diseases or current gastrointestinal symptoms.

### Study protocol

All volunteers fasted overnight [on average 8.7 h, range (7.3–10.1)] before the MRI scan. During the 30 min prior to the MRI scan, they ingested 1 l of 2.5% mannitol solution at regular intervals of 10 min. Mannitol is used routinely in standard MR enterography to provide bowel distension for enteric evaluation.^9^

Scans were acquired with a 3T Philips Ingenia MRI scanner (Philips, Best, Netherlands) in supine position, with subjects positioning their arms at their sides, using a combination of a posterior coil located in the table and an anterior torsocoil covering the entire abdominal region. After initial survey sequences, a coronal single slice two-dimensional balanced fast field echo motility sequence of the bowel was acquired. The slice was positioned to include the terminal ileum if this was visible, together with a good volume of small bowel. The motility scan was acquired during an expiration breath-hold, the volunteers were instructed to hold their breath for approximately 20 s. The scan parameters were: echo time/repetition time: 0.98/1.90 ms, flip angle: 20°, field of view: 400 × 400 mm^2^ [FH (Foot-Head) x LR (Left-Right)], spatial resolution: 2.5 × 2.5 × 10 mm, SENSE factor: 3.1 [RL(Right-Left)], resulting in a temporal resolution of 10 frames per second (fps), also referred to as images per second.

### Motility assessment

Motility data visualization and secondary analysis were performed in MATLAB 2016 (The MathWorks, Natick, MA). This included a graphical user interface, which displayed the dynamic series data sets as a movie as well as a static reference image.

#### Step 1: data set creation

For the assessment of the optimal temporal resolution, all data sets were used at 10 images s^−1^ as acquired from the MRI scanner. Data sets were then retrospectively undersampled to create new data sets at 5, 4, 2, 1, 0.5, 0.25, 0.2, 0.1 fps (the latter meaning 1 image every 10 s).

**Figure 1.**
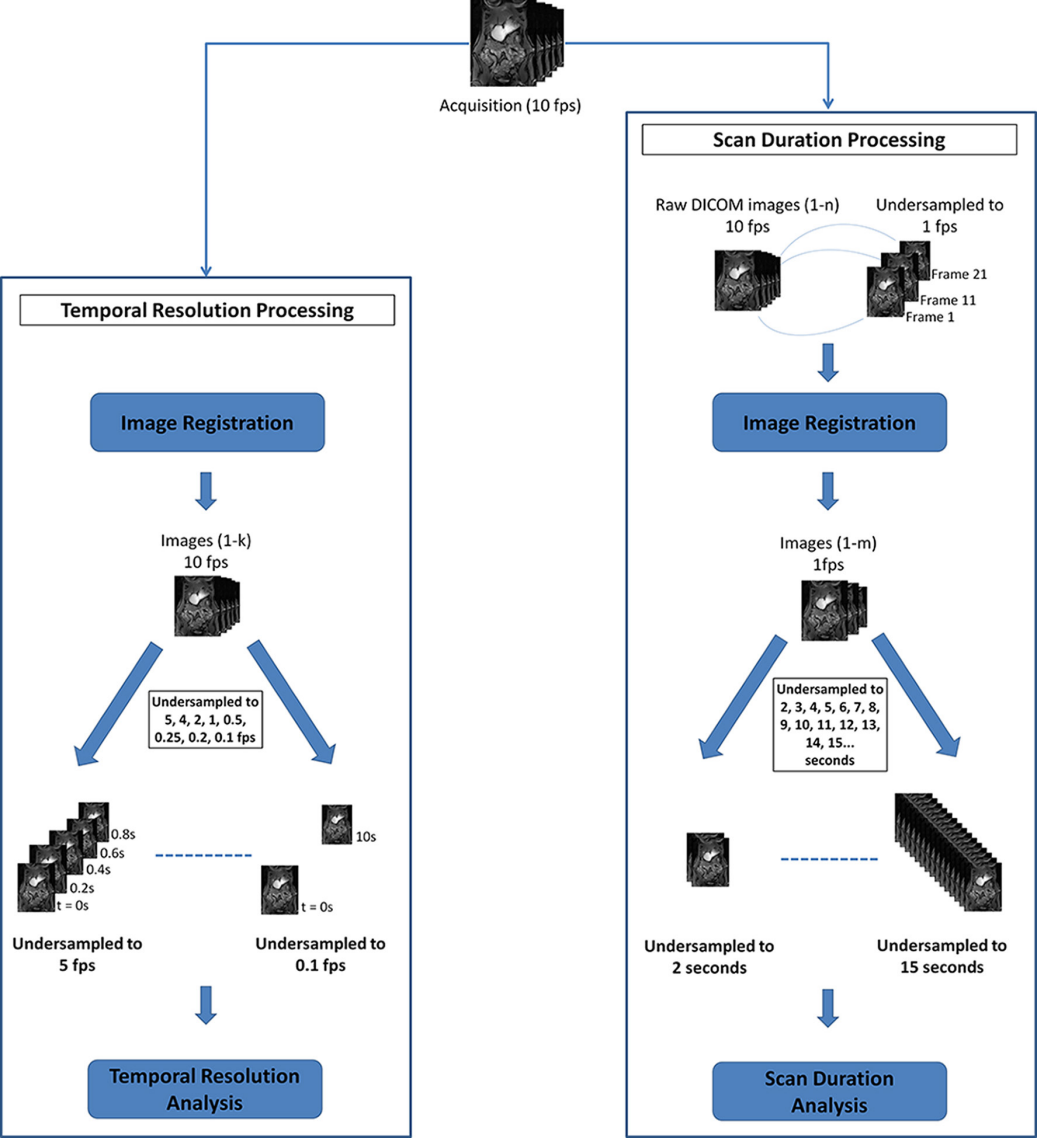
Flow chart of data processing workflow prior to analysis. For the temporal resolution study, the 10 fps dynamic series were registered before being undersampled to 5, 4, 2, 1, 0.5, 0.25, 0.2 and 0.1 fps. For the scan duration study, the 10 fps dynamic series firstly had to be undersampled to 1 fps before being registered. After registration, the data sets were undersampled at 1 s intervals from 2 s to the duration of the data set, *e.g.* 15 s. fps, frames per second.

For the assessment of the optimal duration of data acquisition, every breath-hold dynamics series per subject, acquired at a temporal resolution of 10 images s^−1^, was undersampled to create a data set at a temporal resolution of 1 image s^−1^, consistent with the temporal resolution used in published literature. Figure 1 illustrates the data processing workflow for the two studies.

#### Step 2: image registration

**Figure 2.**
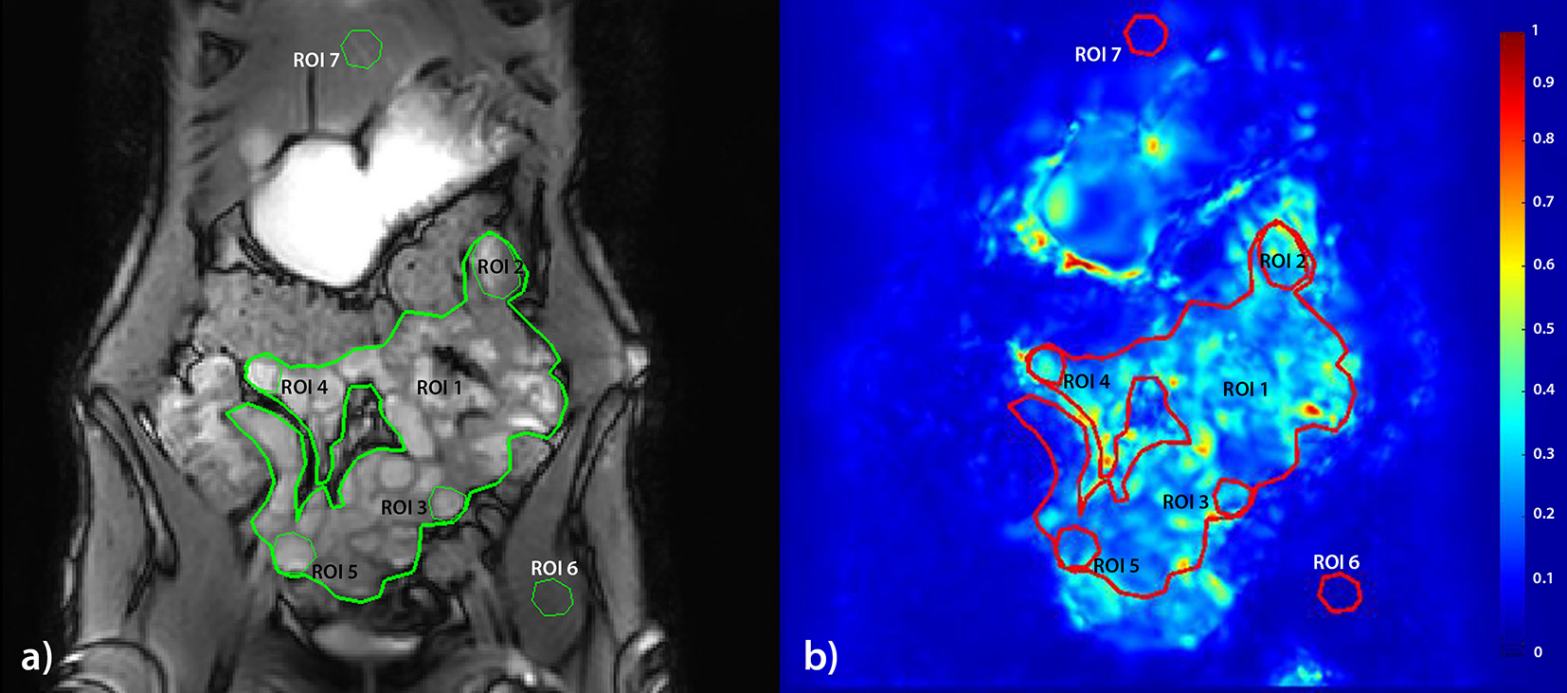
The seven different types of ROIs used in this study; (1) Global small bowel, (2) upper left quadrant of the abdomen, (3) lower left quadrant, (4) upper right quadrant, (5) lower right quadrant, (6) muscle reference, (7) liver reference. The registration target image used for the annotations (a) with motility overlay (b) where blue = low motility and red = high. (ROI, region of interest)

Each created dynamic series described above was registered with an optic flow based technique (GIQuant, Motilent, Ford, UK) developed for bowel motility assessment. The GIQuant software produces a series of deformation fields which can be summarized by taking the standard deviation of each deformation fields’ Jacobian determinant for the time series. This measure is previously validated^13^ as a robust surrogate for motility and can be depicted visually as a color map (Figure 2b) and is henceforth referred to as the motility index.

#### Step 3: calculating the motility index

The motility index is calculated by taking the standard deviation of each deformation fields' Jacobian determinant per pixel. The original Jacobian determinant values were extracted for each time frame.

For the assessment of the optimal temporal resolution, the motility index was calculated for the following temporal resolutions: 10, 5, 4, 2, 1, 0.5, 0.25, 0.2, 0.1 fps. For the assessment of the optimal duration of data acquisition, the motility index was calculated for acquisition times ranging from 2 s up to 20 s in breath-hold data sets, resampled to 1 fps.

#### Step 4: regions of interest (ROIs)

Seven regions of interest (ROIs) were drawn for each subject by CSJ (2 year experience) in consensus with RMG (1 year experience) in all the created data sets. The first ROI was a global ROI, including all visible small bowel followed by four smaller local small bowel ROIs and two reference ROIs, in the liver and a hip muscle (Figure 2). The local ROIs were drawn in four quadrants with upper left, upper right, lower left and lower right of the small bowel (Figure 2). ROIs were individualized according to the anatomy of the volunteer’s bowel volume but were placed in these four quadrants in each volunteer. The ROI for the lower right quadrant was placed in the Ileum, if the terminal ileum was visible the ROI was placed here. The other local ROIs were placed in the jejunum.

The control ROIs were drawn in liver and hip muscle since the pixels in these locations should not change shape or size and therefore, the motility index should be consistent at different temporal resolutions and scan durations.

#### Step 5: motility analysis

The motility index was generated for all ROIs at all tested temporal resolutions and scan durations. The mean motility index within each ROI was plotted against the temporal resolution and scan duration for visualization of the robustness of the motility measure. All data were initially plotted and assessed visually by CSJ (2 year experience) and RMG (1 year experience).

The observers visually assessed at 5 s intervals when the mean motility index appeared to be stabilizing for both the plots of scan duration and temporal resolution. Stabilization was defined as the point where there was little change in mean motility index in data points beyond. The difference between the mean motility index at the point of stabilization and the last data point (*i.e.* the longest scan duration or fastest temporal resolution) was expressed as an absolute percentage change. This change indicates the degree of stabilization of the mean motility index and thereby, the robustness of the motility index after a certain scan duration time and at a particular temporal resolution. In case of no apparent stabilization, the protocol for assessing the data sets was to triplicate the data set, recalculate the motility index and visually inspect the data sets to look for an explanation.

## Results

No adverse effects were observed in the six healthy volunteers [3 females, median age 22 (range 21–25)] The acquisition resulted in breath-holds ranging from 15 to 21 s depending on the subjects ability to hold their breath, see the supplementary video 1 for a resulting breath-hold dynamic MRI. .

### Assessment of optimal temporal resolution

**Figure 3.**
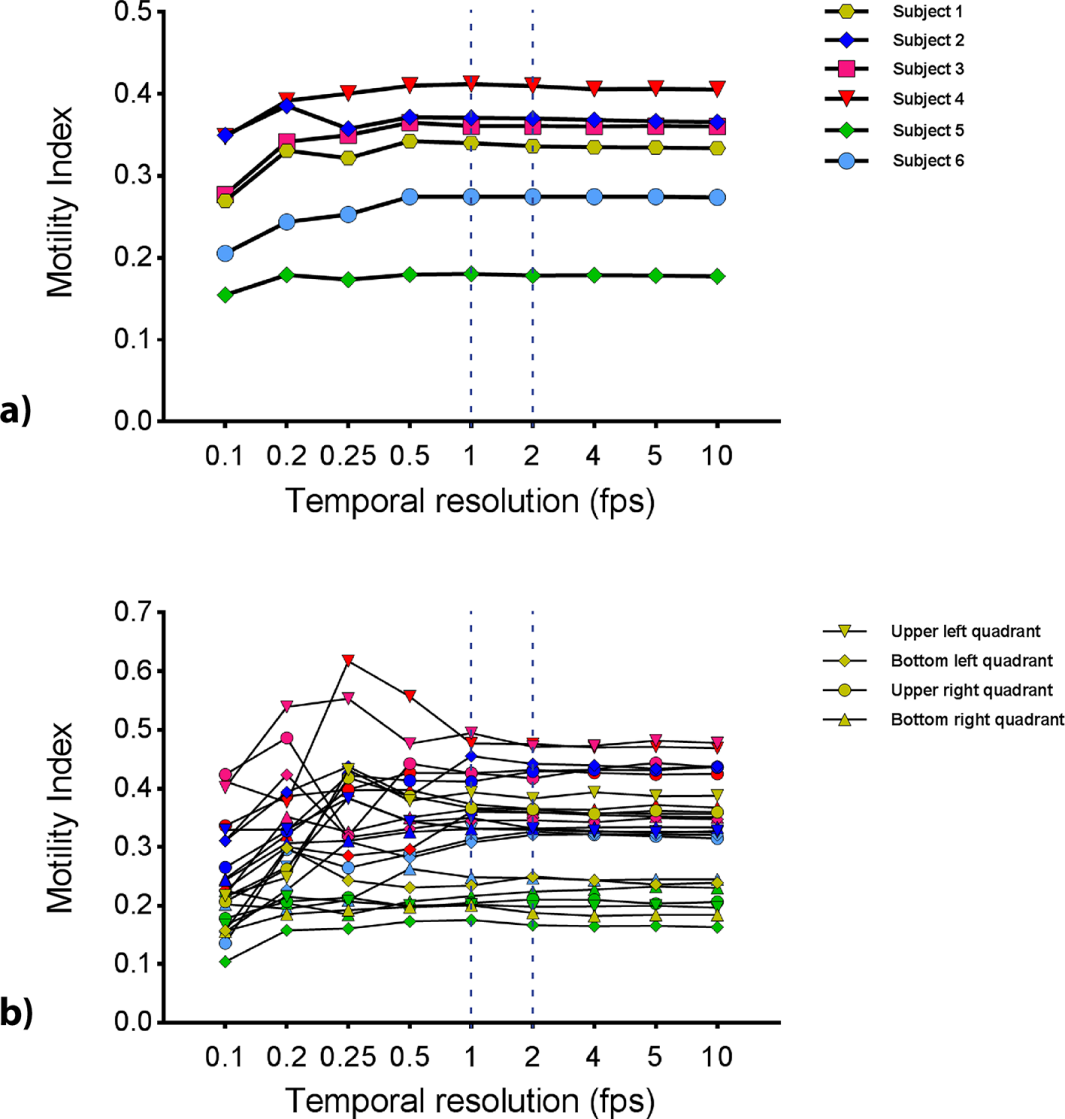
Mean motility index from breath-hold scans for (a) the global small bowel ROI at different temporal resolutions and (b) the quadrant ROIs (ROIs 2–5) calculated at different temporal resolutions. The dotted lines mark 1 and 2fps where the mean motility index appears to be stabilizing and are therefore, the temporal resolutions selected for further analysis. fps, frames per second; ROIs, regions of interest.

Visual inspection of the plot of mean motility index values for each ROI against different temporal resolutions suggested the motility index stabilized at a temporal resolution of 1 fps for both global and local ROIs (Figure 3a,b).

Temporal resolutions of 1 and 2 fps were, therefore selected to assess the stabilization of the mean motility index in comparison to the 10 fps data point (Figure 3).

Table 1 shows the median and range of absolute percentage changes across all subjects for the motility index at temporal resolutions of 1 and 2 fps, in comparison to 10 fps. Global ROI median percentage changes were generally smaller than for the local (quadrant) ROIs.

###  Assessment of optimal duration of data acquisition (scan duration)

**Table 1.** Median and range of the mean motility index absolute percentage change of both global and local ROIs based on selected temporal resolution in breath-hold at 1 and 2 fps

	Global (% change)			Local (% change)		
Median (%)	Range (%)		Median (%)	Range (%)	
Min.	Max.	Min.	Max.
1 fps	1.4	0.1	1.8	1.9	0.3	8.0
2 fps	0.6	0.2	1.2	1.4	0.1	4.5

fps, frames per second; ROIs, regions of interest.

**Figure 4.**
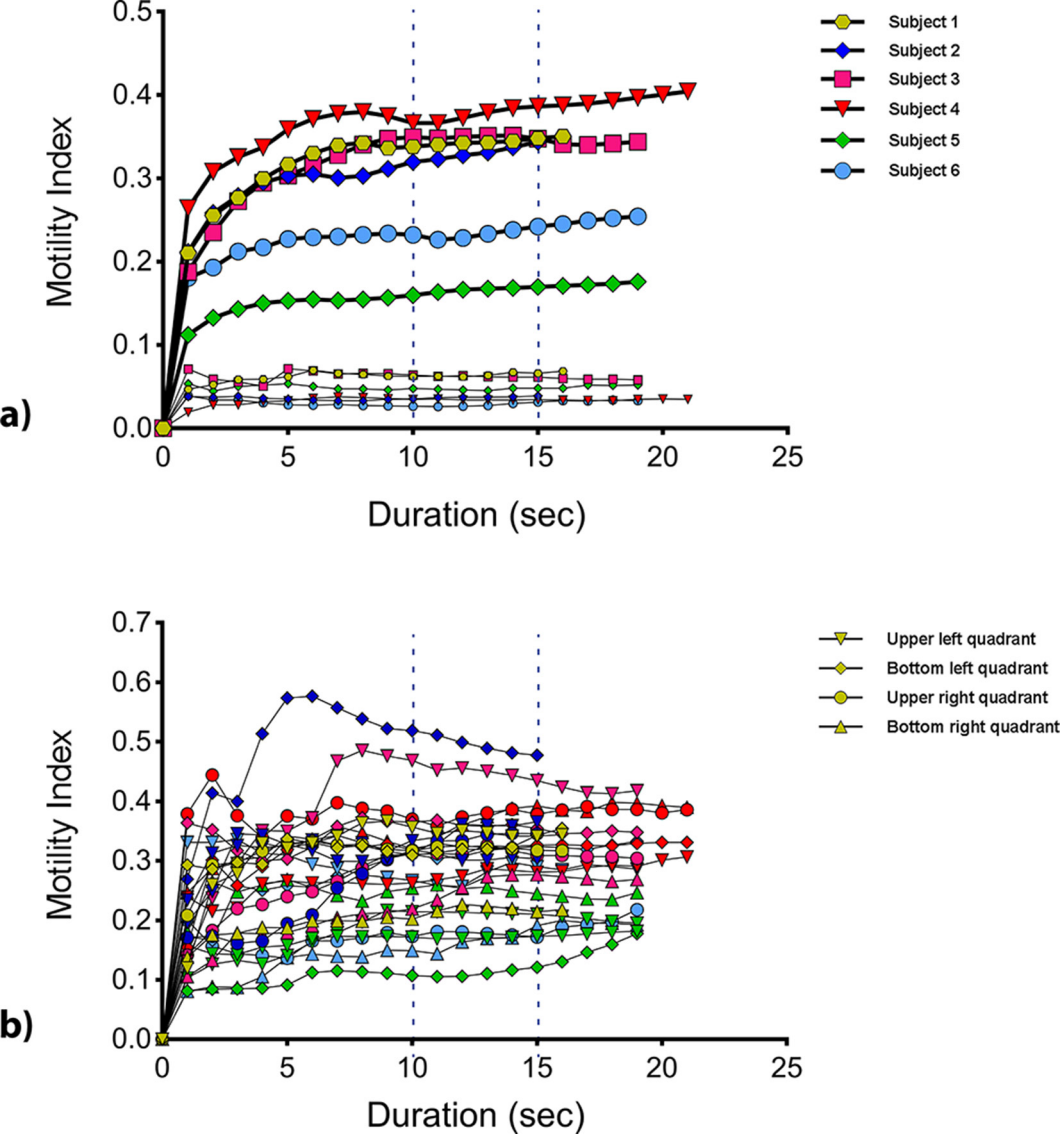
Mean motility index from breath-hold scans for (a) the global ROI and the control ROI’s in muscle at different scan durations and (b) the quadrant ROIs (ROIs 2–5) calculated at different durations. The dotted lines mark 10 and 15 s, where the mean motility index appears to be stabilizing and are therefore, the scan durations selected for further analysis. ROI, region of interest.

Visual inspection of the plot of mean motility index values for each ROI against scan duration during breath-hold suggested the motility index stabilized at a scan duration of 10 s or more for both global and local ROIs (Figure 4a,b).

Based on visual assessment of the breath-hold data, scan duration times at 5 s intervals (10 and 15 s) were selected to assess the stabilization of the mean motility index against the full breath-hold. A scan duration of 5 s was not selected because there was no apparent stabilization in the first 5 s.

Table 2 shows the median and range of absolute percentage changes across all subjects for the motility index at scan durations of 10 and 15 s, in comparison to the full breath-hold durations. The stabilization of the motility index in this assessment demonstrated less stabilization than for temporal resolution, with some data sets characterized by a gradual increase in the mean motility index. Two further tests were performed to understand the source of this climbing mean motility index over time.

**Table 2.** Median and range of the mean motility index absolute percentage change of both global and local ROIs based on selected time points in breath-hold (10 and 15 s)

	**Global (% change)**			**Local (% change)**		
Median (%)	Range (%)		Median (%)	Range (%)	
Min	Max	Min	Max
10 s	8.6	1.6	10.3	7.9	0.0	65.5
15 s	2.8	0.8	5.1	1.7	0.2	47.1

ROIs, regions of interest.

**Figure 5.**
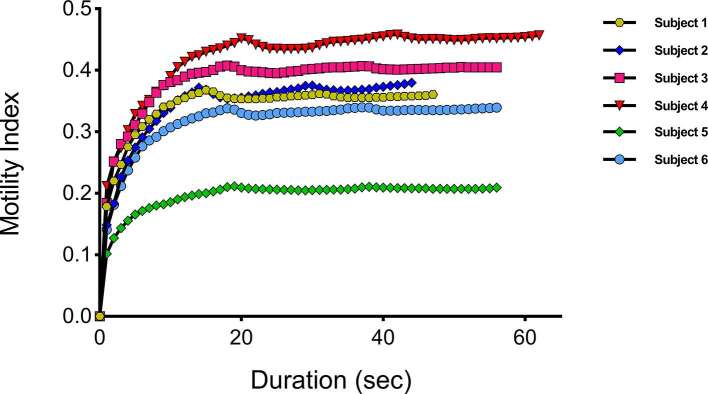
Mean motility index from tripled breath-hold data sets for the global small bowel ROI calculated at different temporal resolutions. ROI, region of interest.

**Figure 6.**
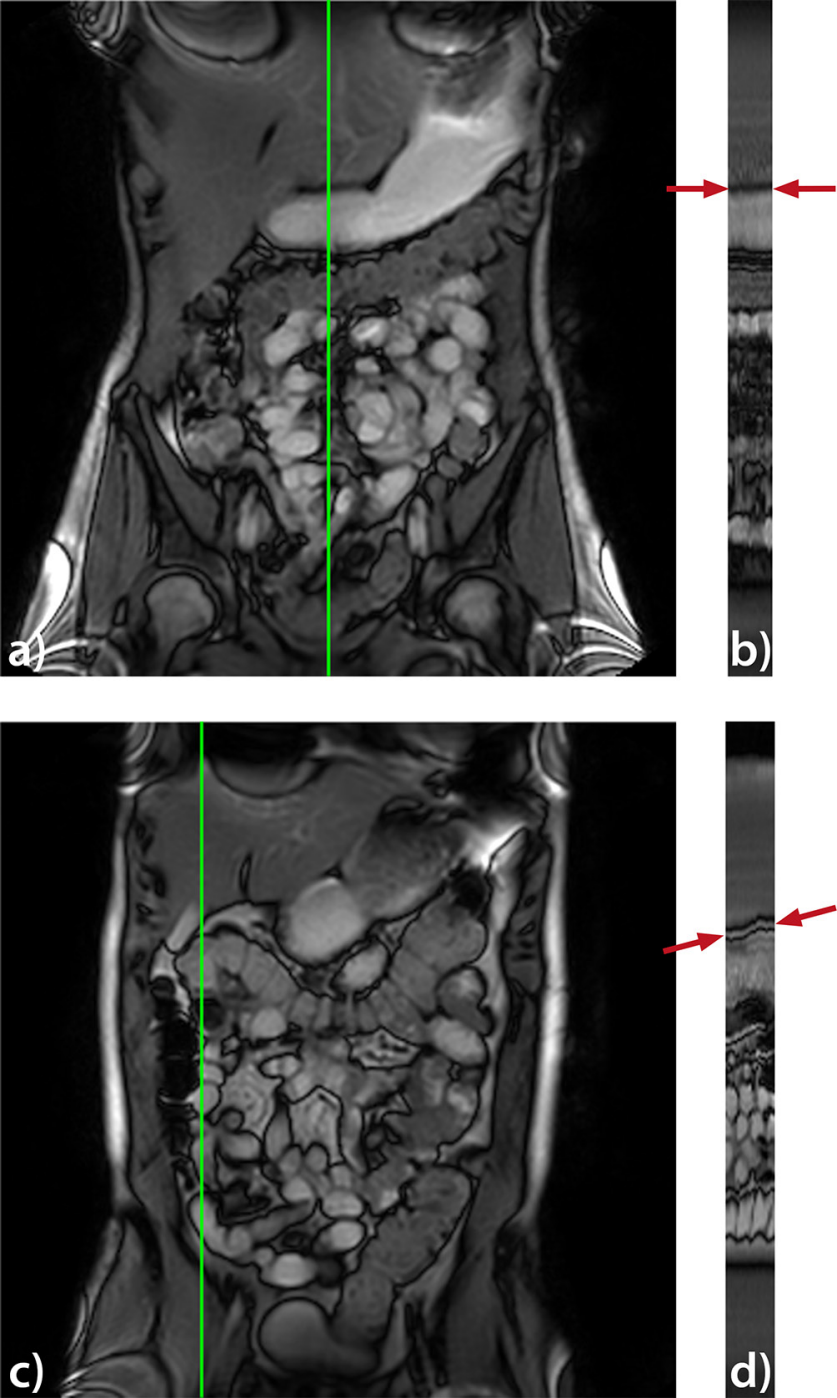
Reference frame from a dynamic data set of a good (a) and an imperfect (c) breath-hold. Image (b and d) shows a cross-section along the temporal direction located at the vertical line indicated in the reference frames (a, c). The cross-section in (d) shows an upwards trend in the positioning of the small bowel (see arrows).

1. By triplicating the breath-hold data set and registering and recalculating the global ROIs, we evaluated the registration algorithm as a potential source of the rising mean motility index. Figure 5 visualizes this additional test and shows that the mean motility index stabilizes as expected and refuting the algorithm itself as the source of this trend.
2. We visually re-assessed the dynamic data sets with a view to establishing a physiological cause of the increasing motility score. Here, we identified three imperfect breath-holds in our data set, showing a slight upwards trend in the positioning of the small bowel over time (Figure 6), despite the breath-hold acquisition. Data sets, where this was not present. did not show the rising mean motility index suggesting that more movement was present in the dynamic data set aside from the bowel motility, producing this artifact.

### Control ROI

Additionally the control ROIs in the liver and hip muscle exhibited very low motility with a mean motility index value below 0.1 and 0.2 respectively indicating very low or no motility occurring at these locations over the duration of an acquisition (Figure 4a).

## Discussion

The purpose of this prospective study was to provide evidence to define the required minimum temporal resolution and scan duration for consistent, quantitative measurements of small bowel motility with dynamic MRI using the motility index. Our data suggest that for a breath-hold scan a temporal resolution of at least 1 image s^−1^ and scan duration of 15 s is necessary for consistent measurements of small bowel motility quantified using a validated registration technique.

The first question we addressed with this study was: at what temporal resolution do we observe consistent motility indices in the small intestine? Too fast and we could oversample the data resulting largely in practical inconveniences, *e.g.* creating an unnecessarily large amount of data for calculations, causing longer post-processing times and with cost implications for data storage. More concerning is acquiring data too slowly leading to undersampling or aliasing motility and potentially generating spurious motility indices with no true physiological meaning.

The temporal resolution of dynamic sequences used in previous research largely ranges around 1 images^-1^ due to adaption of sequences available on clinical systems.^3–7,11,12,15,17,19–22^ These images have a good signal to noise ratio, can be performed rapidly and are practicable for clinical use. Our understanding of gastrointestinal physiology suggests that the small bowel routinely contracts between 9 and 12 times min^−1^^18 ^(0.15–0.2 Hz) suggesting that 1 image s^−1^ (equivalent to 1 Hz) should adequately capture the peristaltic cycle, therefore supporting the practical advantages of the dynamic sequences used in previous research. Reassuringly, by iteratively undersampling a high temporal resolution sequence (10 images s^−1^), we demonstrate in this study that quantification of bowel motility stabilizes at a temporal resolution of 1 fps, with little change at temporal resolutions greater than this.

In support, the median absolute percentage change in motility across a range of ROIs size and positions remained below 2%, when comparing values from 1 fps to much higher temporal resolutions. Such data are welcome, as image acquisition at 1 fps is easily achievable on most 1.5–3 T MRI platforms, with most vendors supplying dynamic sequences as standard. Indeed, acquisition at 2 fps is also possible with only minor sequence modifications. To place this variance in context, a motility study comparing a placebo * vs * a neostigmine stimulant, showed a 22% increase and comparing a placebo * vs * butylscopolamine showed 57% decrease in the mean motility index.^14^ In a Crohn’s study comparing inflamed to non-inflamed terminal ileum, an increase of 95% was observed.^23^ In this study for quadrant ROIs, the median percentage change fell from 1.9% at 1 fps to 1.4% at 2 fps. For global ROIs, the median percentage fell from 1.4% at 1 fps to 0.6% at 2 fps. We feel that this difference is insignificant compared to the differences observed in previous studies however might be important to consider if the potential effect size of future investigations is small.

The sequence used in this study, single-slice rapid acquisition with a flip angle of 20°, is similar to the acquisitions used in previous work imaging multiple slices at a lower temporal resolution.^4, 12,24^ The image quality was adequate for registration and the quality of the registration was assessed visually by playing the frames as a movie. The movie displayed the propagation of the ROIs drawn on the reference frame through all the frames, with the correct registration evident from the alignment of the bowel walls in each of the frames.

The second, and perhaps more difficult question addressed in this study is the duration of the scan. Akin to chosen values of temporal resolution, most researchers to date have been guided by clinical practicalities when choosing their scan duration. Prolonged acquisitions of 5 min or greater are impractical in most busy clinical departments and breath-hold imaging is rapid, practical and limits through plane motion artifacts.

In this study, we investigated the effect of adding additional images to breath-hold data series by modeling prolongation of acquisition protocols ranging from 2 to 22 s. Specifically, we evaluated if the mean motility index would change as new data were added to the series. Reassuringly, we found that for most breath-hold data sets, acquisitions of less than 20 s were adequate and stabilization in the motility index was visualized although a persistent “creep” was observed in several cases. For small local ROIs, the motility did seem more variable, the median percentage change fell below 10% at 10 s in breath-hold (7.9%), likely due to inherent variation in bowel contractility in smaller bowel regions. Data were more consistent for the larger global ROIs, the median percentage falls below 10% at 10 s in breath-hold (8.6%), likely due to averaging of motility over the full bowel volume. For both global and local ROIs, the median percentage changes falls below 5% at 15 s in breath-hold (global = 2.8%, local = 1.7%).

As reported in the results, we observed a general, positive trend in the mean motility index with the increasing number of time points. Our concerns were that either there might be a systematic bias in the algorithm or that inherent physiological variation was leading to an evolution of the motility score and that, a 20 s observation was insufficient to observe small bowel motility. We conducted two further experiments to resolve the potential cause of this effect. We triplicated the same breath-hold motility data for each subject before recalculating the mean motility index and reassuringly found that the mean motility index plot stabilized. Allowing us to reject the registration algorithm itself being the source of this upward mean motility index trend. Had there been a cumulative error, we would have seen an increasing value over the 60 time points.

Second, we noticed there was a slight upwards trend in the positioning of the small bowel between the first and last frame in the dynamic series when reviewed as a cine loop. This “jump” was particularly pronounced where the upward inflection in the mean motility index plot was seen and absent where the mean motility index values flattened out. This result suggests that the quality of the breath-hold is important as well beyond the simple use of breath-hold. Going forward, the presence of the upwards trend is relatively simple to check for and potentially avoid with careful communication with the subject. Further, this artifact appears to be a bias which might be corrected *post-hoc* that subtly alters the mean motility index value, this change is below the effect size seen in clinical studies and we do not, therefore, feel this impacts existing published research. It will be useful to examine this phenomenon in free-breathing examinations to assure that there is no bias introduced in the mean motility index by the breathing in these data sets. However, since a different registration algorithm is used to calculate the motility index in free-breathing data sets,^24, 25^ breath-hold and free-breathing cannot be directly compared.

This study does have limitations. It is important to interpret our data in the context of the registration algorithm used to generate the motility index. Although well validated in healthy volunteers and patient groups^4,12–14,17^ the metric, described as the standard deviation of the deformation fields’ Jacobian determinant, is a surrogate measure of motility only, rather than a measure of a defined, physiological action (*i.e.* peristalsis). The metric captures information on how the bowel deforms and, by taking the standard deviation, temporal information on the frequency of contractions is lost. That is, if a bowel loop underwent multiple rapid contractions of equal amplitude, the same motility index would be recorded as if it only underwent one large contraction. There was little gain in accelerating the acquisition to faster than 1 s^-1^, although this may be useful for small ROIs. The literature currently reports a range of diameter based measurements (*e.g.* contractions per minute),^6, 11,26,27^ but as we had relatively short data sets in terms of minutes, we did not feel we were able to do a thorough investigation of such metrics here. This study only explored one motility metric, conversely, using a frequency metric like “contractions per minute” may not, by definition, be robust under these circumstances.

Additionally, the sample size of six subjects is relatively small. Nevertheless, we feel that these data are still representative of subjects seen in the clinical setting, given the known heterogeneity in bowel motility described previously by Menys et al^12^ collected in a cohort of 20 patients (four ROIs per patient).

Another consideration is the underlying bowel physiology in terms of fed and fasted motility patterns. Clinically, patients are required to fast prior to small bowel enterography and then ingest up to 2 l of a contrast solution to distend the bowel for visualization purposes. Although the solution has almost no caloric component and therefore should not, in theory, drive fed motion patterns, a marked increase in motility is often seen in the prepared bowel and one might infer that they are seeing segmentation and peristalsis.^28^ This likely prokinetic effect of, in this case, mannitol seems to drive and elevate motility homogeneously along the bowel resulting in relatively clustered data. The scan duration may need to be longer in cases where motility is not stimulated and this should be investigated in further studies.

## Conclusion

In summary, this study shows that a temporal resolution of 1 image s^−1^ over a scan duration of 15 s in breath-hold is sufficient to obtain robust measurements of small bowel motility from MRI when quantified using optic flow registration techniques.
